# Hemiaqua­bis(2-fluoro­benzoato-κ^2^ *O*,*O*′)bis­(1,10-phenanthroline-κ^2^ *N*,*N*′)lead(II) dihydrate

**DOI:** 10.1107/S1600536809027524

**Published:** 2009-07-18

**Authors:** Su-Fang Ye, Bi-Song Zhang

**Affiliations:** a College of Materials Science and Chemical Engineering, Jinhua College of Profession and Technology, Jinhua, Zhejiang 321017, People’s Republic of China

## Abstract

In the title compound, [Pb(C_7_H_4_FO_2_)_2_(C_12_H_8_N_2_)_2_(H_2_O)_0.5_]·2H_2_O, the Pb^II^ atom is coordinated by four N atoms from two bidentate chelating 1,10-phenanthroline (phen) ligands, four O atoms from two 2-fluoro­benzoate ligands and a half-occupied water mol­ecule in an irregular coordination geometry. One carboxyl­ate O atom and two F atoms are each disordered over two sites with occupancy factors of 0.558 (6) and 0.442 (6). The two crystallographically independent phen ligands are co-planar [dihedral angle 0.0 (2)°]. Centroid–centroid distances of 3.659 (7) and 3.687 (7) Å indicate π–π stacking inter­actions between neighboring phen ligands. In the crystal, O—H⋯O, C—H⋯F and C—H⋯O hydrogen bonds link the complex mol­ecules and uncoordinated water mol­ecules into a supra­molecular network.

## Related literature

For other complexes with a 2(or 4)-fluoro­benzoate ligand, see: Zhang *et al.* (2005 ▶). For related structures, see: Zhang (2004 ▶, 2005 ▶, 2006*a* ▶,*b* ▶,*c* ▶).
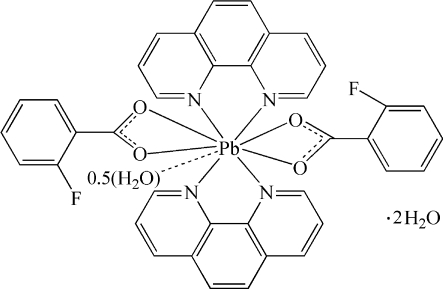


## Experimental

### 

#### Crystal data

[Pb(C_7_H_4_FO_2_)_2_(C_12_H_8_N_2_)_2_(H_2_O)_0.5_]·2H_2_O
*M* *_r_* = 890.84
Triclinic, 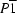

*a* = 9.833 (2) Å
*b* = 11.568 (2) Å
*c* = 15.766 (3) Å
α = 81.11 (3)°
β = 77.23 (3)°
γ = 86.20 (3)°
*V* = 1727.0 (6) Å^3^
*Z* = 2
Mo *K*α radiation
μ = 4.95 mm^−1^
*T* = 290 K
0.34 × 0.19 × 0.16 mm

#### Data collection

Bruker SMART APEX CCD diffractometer
Absorption correction: multi-scan (*SADABS*; Sheldrick, 1996 ▶) *T*_min_ = 0.330, *T*_max_ = 0.448
28426 measured reflections
10232 independent reflections
7916 reflections with *I* > 2σ(*I*)
*R*_int_ = 0.028

#### Refinement

*R*[*F* ^2^ > 2σ(*F* ^2^)] = 0.032
*wR*(*F* ^2^) = 0.078
*S* = 1.02
10232 reflections
466 parameters
3 restraints
H-atom parameters constrained
Δρ_max_ = 1.33 e Å^−3^
Δρ_min_ = −0.77 e Å^−3^

### 

Data collection: *SMART* (Bruker, 2007 ▶); cell refinement: *SAINT* (Bruker, 2007 ▶); data reduction: *SAINT*; program(s) used to solve structure: *SHELXS97* (Sheldrick, 2008 ▶); program(s) used to refine structure: *SHELXL97* (Sheldrick, 2008 ▶); molecular graphics: *SHELXTL* (Sheldrick, 2008 ▶); software used to prepare material for publication: *SHELXTL*.

## Supplementary Material

Crystal structure: contains datablocks I, global. DOI: 10.1107/S1600536809027524/hy2206sup1.cif

Structure factors: contains datablocks I. DOI: 10.1107/S1600536809027524/hy2206Isup2.hkl

Additional supplementary materials:  crystallographic information; 3D view; checkCIF report

## Figures and Tables

**Table 1:** Selected bond lengths (Å)

Pb1—O1	2.957 (8)
Pb1—O1′	2.866 (9)
Pb1—O2	2.631 (3)
Pb1—O3	2.575 (3)
Pb1—O4	2.570 (3)
Pb1—N1	2.796 (3)
Pb1—N2	2.656 (3)
Pb1—N3	2.768 (3)
Pb1—N4	2.906 (3)
Pb1—O7*W*	2.965 (7)

**Table 2:** Hydrogen-bond geometry (Å, °)

*D*—H⋯*A*	*D*—H	H⋯*A*	*D*⋯*A*	*D*—H⋯*A*
O5*W*—H5*WA*⋯O2	0.85	1.98	2.796 (4)	162
O5*W*—H5*WB*⋯O6*W*	0.85	1.97	2.757 (5)	153
O6*W*—H6*WA*⋯O5*W*^i^	0.85	1.98	2.809 (6)	163
O6*W*—H6*WB*⋯O4	0.85	1.97	2.818 (3)	175
O6*W*—H6*WA*⋯O5*W*^i^	0.85	1.98	2.809 (6)	163
O7*W*—H7*WA*⋯O1′	0.85	1.98	2.496 (5)	118
O7*W*—H7*WB*⋯O1′^ii^	0.85	1.99	2.565 (2)	124
C8—H8⋯F1^iii^	0.93	2.54	3.310 (7)	141
C30—H30⋯F1′^ii^	0.93	2.50	3.032 (12)	115
C29—H29⋯O3^iv^	0.93	2.46	3.311 (5)	153

Symmetry codes: (i) 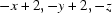; (ii) 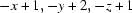; (iii) 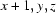; (iv) 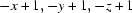.

**Fig. 1.**
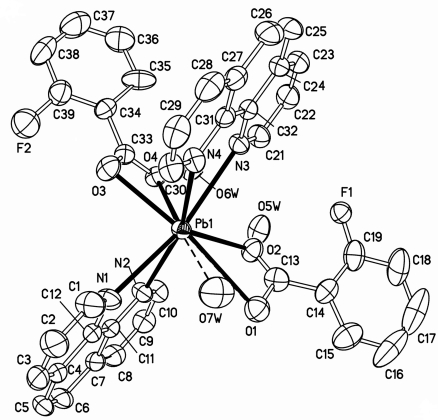
Molecular structure of the title compound. Displacement ellipsoids are drawn at the 50% probability level. H atoms have been omitted for clarity. Dashed line shows the bond between Pb1 and half-occupied O7W.

**Fig. 2.**
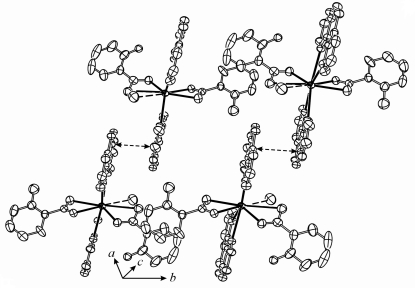
The π–π stacking interactions (dashed double arrows), with the centroid–centroid distances of 3.659 (7) and 3.687 (7) Å.

**Fig. 3.**
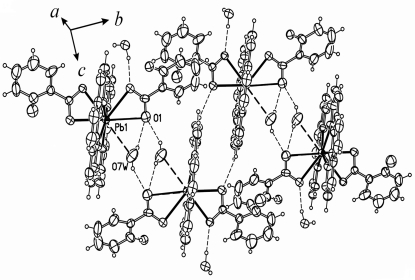
The hydrogen bonds (thin dashed lines) in the title compound.

## supplementary crystallographic information

### Comment

We have prepared the title complex by the hydrothermal reaction of freshly prepared PbCO_3_ with 1,10-phenanthroline (phen) and 2-fluorobenzoic acid in CH_3_OH/H_2_O, and report here its crystal structure (Fig. 1). The title compound has a structure similar to those of complexes with halobenzoate ligands, X–C_6_H_4_COO^-^, where X is F, Cl, Br and I (Zhang, 2004, 2005, 2006a,b,c; Zhang *et al.*, 2005). The asymmetric unit of the title compound consists of a [Pb(C_7_H_4_FO_2_)_2_(C_12_H_8_N_2_)_2_(H_2_O)_0.5_] complex molecule and two uncoordinated water molecules. The Pb^II^ atom is coordinated by four N atoms from two bidentate chelating phen ligands, four O atoms from two 2-fluorobenzoate ligands and a half-occupied water molecule in an irregular coordination geometry, with Pb—N bond lengths in the range of 2.656 (3) to 2.906 (3)Å and Pb—O bond lengths in the range of 2.570 (3) to 2.965 (7)Å (Table 1). The centroid–centroid distances of 3.659 (7) and 3.687 (7) Å indicate π–π stacking interactions between the neighboring phen ligands (Fig. 2). O—H···O, C—H···F and C—H···O hydrogen bonds are also present (Table 2 and Fig. 3). A combination of the π–π stacking interactions and hydrogen bonds leads to a supramolecular network.

### Experimental

Pb(CH_3_COO)_2_.3H_2_O (0.17 g, 0.45 mmol) was dissolved in appropriate amount of water, and then 1M Na_2_CO_3_ solution was added. PbCO_3_ was obtained by filtration, which was then washed with distilled water for 5 times. The freshly prepared PbCO_3_, phen (0.05 g, 0.25 mmol), 2-fluorobenzoic acid (0.04 g, 0.29 mmol), CH_3_OH/H_2_O (v/v = 1:2, 15 ml) were mixed and stirred for 1.5 h. Subsequently, the resulting cream suspension was heated in a 23 ml Teflon-lined stainless steel autoclave at 423 K for one week. After the autoclave was cooled to room temperature, the solid was filtered off. The resulting filtrate was allowed to stand at room temperature, and evaporation for 3 weeks afforded colorless transparent pillar-like single crystals. Analysis calculated for C_38_H_29_F_2_N_4_O_6.5_Pb: C 51.19, H 3.26, N 6.28%; found: C 51.06, H 3.06, N 6.13%.

### Refinement

The disordered O and F atoms on the ligands were refined isotropically. H atoms on C atoms were positioned geometrically and refined as riding atoms, with C—H = 0.93 Å and with *U*_iso_(H) = 1.2*U*_eq_(C). H atoms of water molecules were located in a difference Fourier map and refined with restraints of O—H = 0.85 (1) Å and U_iso_(H) = 1.5U_eq_(O). The largest peak in the final difference Fourier map is 0.24 Å from atom H35 and the deepest hole is 0.52 Å from atom F2'.

### Crystal data

**Table:**

[Pb(C_7_H_4_FO_2_)_2_(C_12_H_8_N_2_)_2_(H_2_O)_0.5_]·2H_2_O	*Z* = 2
*M**_r_* = 890.84	*F*(000) = 874
Triclinic, *P*1	*D*_x_ = 1.713 Mg m^−^^3^
Hall symbol: -P 1	Mo *K*α radiation, λ = 0.71073 Å
*a* = 9.833 (2) Å	Cell parameters from 8657 reflections
*b* = 11.568 (2) Å	θ = 2.7–30.5°
*c* = 15.766 (3) Å	µ = 4.95 mm^−^^1^
α = 81.11 (3)°	*T* = 290 K
β = 77.23 (3)°	Pillar-like, colorless
γ = 86.20 (3)°	0.34 × 0.19 × 0.16 mm
*V* = 1727.0 (6) Å^3^	

### Data collection

**Table:**

Bruker SMART APEX CCD diffractometer	10232 independent reflections
Radiation source: fine-focus sealed tube	7916 reflections with *I* > 2σ(*I*)
graphite	*R*_int_ = 0.028
φ and ω scans	θ_max_ = 30.5°, θ_min_ = 2.7°
Absorption correction: multi-scan (*SADABS*; Sheldrick, 1996)	*h* = −14→13
*T*_min_ = 0.330, *T*_max_ = 0.448	*k* = −16→16
28426 measured reflections	*l* = −22→22

### Refinement

**Table:**

Refinement on *F*^2^	Primary atom site location: structure-invariant direct methods
Least-squares matrix: full	Secondary atom site location: difference Fourier map
*R*[*F*^2^ > 2σ(*F*^2^)] = 0.032	Hydrogen site location: inferred from neighbouring sites
*wR*(*F*^2^) = 0.078	H-atom parameters constrained
*S* = 1.01	*w* = 1/[σ^2^(*F*_o_^2^) + (0.0433*P*)^2^] where *P* = (*F*_o_^2^ + 2*F*_c_^2^)/3
10232 reflections	(Δ/σ)_max_ = 0.002
466 parameters	Δρ_max_ = 1.33 e Å^−^^3^
3 restraints	Δρ_min_ = −0.77 e Å^−^^3^

### Fractional atomic coordinates and isotropic or equivalent isotropic displacement parameters (Å^2^)

**Table:**

	*x*	*y*	*z*	*U*_iso_*/*U*_eq_	Occ. (<1)
Pb1	0.694779 (12)	0.832399 (10)	0.325534 (7)	0.04599 (5)	
N1	0.8374 (4)	0.7952 (3)	0.4629 (2)	0.0650 (8)	
N2	0.9505 (3)	0.9112 (3)	0.29959 (19)	0.0529 (7)	
N3	0.4998 (3)	0.7983 (3)	0.22999 (19)	0.0535 (7)	
N4	0.4509 (3)	0.6922 (3)	0.4003 (2)	0.0570 (7)	
O1	0.6395 (9)	1.0811 (7)	0.3491 (5)	0.075 (2)*	0.442 (6)
O1'	0.5776 (10)	1.0611 (8)	0.3514 (6)	0.112 (3)*	0.558 (6)
O2	0.6834 (3)	1.0286 (2)	0.21751 (18)	0.0670 (7)	
O3	0.7824 (3)	0.6229 (2)	0.30117 (19)	0.0718 (8)	
O4	0.8193 (3)	0.7548 (2)	0.18316 (16)	0.0564 (6)	
O5W	0.8186 (4)	1.0878 (3)	0.0418 (2)	0.0933 (10)	
H5WA	0.7855	1.0837	0.0967	0.140*	
H5WB	0.8144	1.0180	0.0321	0.140*	
O6W	0.8963 (4)	0.8634 (3)	0.0076 (2)	0.1016 (11)	
H6WA	0.9847	0.8631	−0.0092	0.152*	
H6WB	0.8676	0.8313	0.0603	0.152*	
O7W	0.5357 (7)	0.9161 (8)	0.4888 (6)	0.114 (3)	0.50
H7WA	0.5897	0.9728	0.4679	0.170*	0.50
H7WB	0.5360	0.8824	0.5406	0.170*	0.50
F1	0.4835 (5)	1.1309 (4)	0.1281 (3)	0.0642 (16)*	0.442 (6)
F1'	0.6161 (11)	1.3066 (9)	0.3310 (7)	0.174 (4)*	0.558 (6)
F2	0.9722 (9)	0.4352 (8)	0.2413 (6)	0.1118 (17)*	0.442 (6)
F2'	0.9188 (7)	0.4233 (6)	0.2639 (5)	0.1118 (17)*	0.558 (6)
C1	0.7849 (6)	0.7420 (4)	0.5430 (3)	0.0860 (14)	
H1	0.6973	0.7103	0.5529	0.103*	
C2	0.8501 (7)	0.7302 (5)	0.6125 (3)	0.0926 (15)	
H2	0.8076	0.6915	0.6672	0.111*	
C3	0.9756 (7)	0.7753 (4)	0.6002 (3)	0.0866 (15)	
H3	1.0213	0.7676	0.6467	0.104*	
C4	1.0395 (5)	0.8345 (4)	0.5173 (3)	0.0661 (11)	
C5	1.1716 (5)	0.8861 (4)	0.5000 (4)	0.0812 (14)	
H5	1.2202	0.8820	0.5448	0.097*	
C6	1.2265 (4)	0.9402 (5)	0.4201 (4)	0.0812 (14)	
H6	1.3146	0.9710	0.4099	0.097*	
C7	1.1553 (4)	0.9528 (3)	0.3497 (3)	0.0618 (9)	
C8	1.2079 (4)	1.0116 (4)	0.2665 (4)	0.0762 (13)	
H8	1.2944	1.0457	0.2548	0.091*	
C9	1.1344 (5)	1.0203 (4)	0.2018 (3)	0.0723 (11)	
H9	1.1690	1.0605	0.1461	0.087*	
C10	1.0081 (4)	0.9681 (4)	0.2211 (3)	0.0649 (10)	
H10	0.9593	0.9727	0.1764	0.078*	
C11	1.0242 (3)	0.9021 (3)	0.3638 (2)	0.0516 (8)	
C12	0.9641 (4)	0.8418 (3)	0.4497 (2)	0.0554 (8)	
C13	0.6226 (4)	1.0950 (4)	0.2690 (2)	0.0671 (10)	
C14	0.5605 (4)	1.2106 (4)	0.2336 (3)	0.0667 (10)	
C15	0.5581 (7)	1.3120 (6)	0.2687 (4)	0.1057 (18)	
H15	0.5976	1.3075	0.3176	0.127*	0.442 (6)
C16	0.5055 (10)	1.4165 (7)	0.2405 (7)	0.162 (4)	
H16	0.5106	1.4823	0.2668	0.195*	
C17	0.4438 (12)	1.4207 (9)	0.1708 (8)	0.189 (6)	
H17	0.4027	1.4909	0.1500	0.227*	
C18	0.4406 (8)	1.3262 (7)	0.1311 (5)	0.134 (3)	
H18	0.3991	1.3324	0.0830	0.161*	
C19	0.4987 (5)	1.2199 (5)	0.1615 (3)	0.0873 (15)	
H19	0.4965	1.1549	0.1337	0.105*	0.558 (6)
C21	0.5238 (5)	0.8488 (4)	0.1454 (3)	0.0667 (10)	
H21	0.6039	0.8915	0.1229	0.080*	
C22	0.4337 (5)	0.8395 (4)	0.0906 (3)	0.0769 (12)	
H22	0.4534	0.8762	0.0327	0.092*	
C23	0.3175 (5)	0.7773 (4)	0.1216 (3)	0.0787 (13)	
H23	0.2570	0.7707	0.0850	0.094*	
C24	0.2883 (4)	0.7231 (4)	0.2079 (3)	0.0644 (10)	
C25	0.1654 (5)	0.6569 (5)	0.2460 (4)	0.0849 (14)	
H25	0.1015	0.6494	0.2119	0.102*	
C26	0.1415 (4)	0.6059 (5)	0.3298 (4)	0.0864 (14)	
H26	0.0612	0.5635	0.3526	0.104*	
C27	0.2356 (4)	0.6151 (3)	0.3845 (3)	0.0640 (10)	
C28	0.2154 (5)	0.5624 (4)	0.4730 (4)	0.0818 (14)	
H28	0.1364	0.5190	0.4978	0.098*	
C29	0.3075 (5)	0.5735 (4)	0.5217 (3)	0.0794 (13)	
H29	0.2946	0.5380	0.5799	0.095*	
C30	0.4237 (4)	0.6403 (4)	0.4825 (3)	0.0700 (11)	
H30	0.4869	0.6489	0.5170	0.084*	
C31	0.3572 (3)	0.6795 (3)	0.3515 (2)	0.0508 (7)	
C32	0.3842 (3)	0.7350 (3)	0.2611 (2)	0.0519 (8)	
C33	0.8114 (3)	0.6510 (3)	0.2198 (2)	0.0519 (8)	
C34	0.8350 (4)	0.5560 (3)	0.1620 (3)	0.0624 (9)	
C35	0.7819 (6)	0.5800 (5)	0.0821 (3)	0.1034 (18)	
H35	0.7385	0.6508	0.0647	0.124*	
C36	0.8019 (8)	0.4873 (7)	0.0339 (5)	0.130 (2)	
H36	0.7718	0.4983	−0.0188	0.156*	
C37	0.8619 (9)	0.3820 (7)	0.0576 (6)	0.133 (3)	
H37	0.8681	0.3236	0.0224	0.160*	
C38	0.9122 (7)	0.3607 (5)	0.1308 (5)	0.114 (2)	
H38	0.9570	0.2897	0.1463	0.137*	
C39	0.8944 (5)	0.4497 (4)	0.1824 (3)	0.0824 (12)	

### Atomic displacement parameters (Å^2^)

**Table:**

	*U*^11^	*U*^22^	*U*^33^	*U*^12^	*U*^13^	*U*^23^
Pb1	0.05017 (8)	0.04824 (8)	0.04002 (7)	−0.00731 (5)	−0.01187 (5)	−0.00214 (5)
N1	0.084 (2)	0.066 (2)	0.0500 (17)	−0.0199 (17)	−0.0252 (16)	0.0001 (14)
N2	0.0529 (15)	0.0569 (17)	0.0524 (16)	−0.0064 (13)	−0.0191 (13)	−0.0061 (13)
N3	0.0539 (16)	0.0579 (17)	0.0510 (16)	−0.0040 (13)	−0.0176 (13)	−0.0055 (13)
N4	0.0508 (16)	0.0616 (19)	0.0534 (17)	−0.0048 (13)	−0.0081 (13)	0.0045 (14)
O2	0.0816 (18)	0.0576 (16)	0.0581 (15)	0.0021 (13)	−0.0133 (13)	−0.0013 (12)
O3	0.095 (2)	0.0565 (16)	0.0581 (16)	−0.0063 (14)	−0.0101 (14)	0.0023 (12)
O4	0.0677 (15)	0.0500 (14)	0.0494 (13)	−0.0075 (11)	−0.0086 (11)	−0.0034 (11)
O5W	0.113 (2)	0.082 (2)	0.0669 (18)	0.0181 (18)	0.0005 (17)	0.0069 (15)
O6W	0.130 (3)	0.080 (2)	0.074 (2)	0.008 (2)	0.006 (2)	0.0087 (17)
O7W	0.086 (5)	0.142 (7)	0.112 (6)	−0.007 (5)	0.002 (4)	−0.048 (5)
C1	0.117 (4)	0.078 (3)	0.065 (3)	−0.028 (3)	−0.029 (3)	0.009 (2)
C2	0.144 (5)	0.083 (3)	0.055 (2)	−0.006 (3)	−0.041 (3)	0.004 (2)
C3	0.133 (4)	0.072 (3)	0.069 (3)	0.026 (3)	−0.055 (3)	−0.019 (2)
C4	0.088 (3)	0.053 (2)	0.071 (2)	0.020 (2)	−0.042 (2)	−0.0250 (19)
C5	0.077 (3)	0.090 (3)	0.101 (4)	0.027 (2)	−0.056 (3)	−0.047 (3)
C6	0.053 (2)	0.094 (3)	0.115 (4)	0.006 (2)	−0.039 (2)	−0.045 (3)
C7	0.0517 (19)	0.062 (2)	0.080 (3)	0.0078 (16)	−0.0221 (18)	−0.028 (2)
C8	0.048 (2)	0.078 (3)	0.103 (4)	−0.0112 (19)	−0.006 (2)	−0.025 (3)
C9	0.067 (2)	0.072 (3)	0.072 (3)	−0.011 (2)	−0.004 (2)	−0.004 (2)
C10	0.060 (2)	0.076 (3)	0.057 (2)	−0.0078 (19)	−0.0125 (17)	−0.0020 (19)
C11	0.0530 (18)	0.0459 (18)	0.061 (2)	0.0070 (14)	−0.0187 (16)	−0.0182 (15)
C12	0.068 (2)	0.0455 (18)	0.061 (2)	0.0095 (16)	−0.0291 (18)	−0.0156 (15)
C13	0.081 (3)	0.074 (3)	0.0417 (18)	−0.012 (2)	−0.0091 (18)	0.0050 (17)
C14	0.067 (2)	0.060 (2)	0.061 (2)	−0.0025 (18)	0.0112 (19)	−0.0094 (18)
C15	0.114 (4)	0.096 (4)	0.101 (4)	−0.006 (3)	0.006 (3)	−0.035 (3)
C16	0.155 (8)	0.069 (4)	0.225 (11)	−0.004 (5)	0.062 (7)	−0.050 (6)
C17	0.191 (10)	0.097 (6)	0.227 (13)	0.070 (7)	0.024 (9)	0.003 (7)
C18	0.153 (6)	0.114 (5)	0.122 (5)	0.057 (5)	−0.036 (5)	0.007 (4)
C19	0.087 (3)	0.077 (3)	0.084 (3)	0.013 (2)	−0.011 (3)	0.016 (3)
C21	0.077 (3)	0.075 (3)	0.050 (2)	−0.004 (2)	−0.0205 (19)	−0.0026 (18)
C22	0.103 (3)	0.082 (3)	0.051 (2)	0.011 (3)	−0.032 (2)	−0.011 (2)
C23	0.089 (3)	0.079 (3)	0.085 (3)	0.012 (2)	−0.049 (3)	−0.027 (2)
C24	0.059 (2)	0.063 (2)	0.082 (3)	0.0078 (17)	−0.030 (2)	−0.028 (2)
C25	0.060 (2)	0.085 (3)	0.123 (4)	−0.008 (2)	−0.031 (3)	−0.035 (3)
C26	0.050 (2)	0.087 (3)	0.126 (4)	−0.015 (2)	−0.009 (3)	−0.034 (3)
C27	0.0493 (19)	0.050 (2)	0.089 (3)	−0.0029 (15)	−0.0022 (19)	−0.0144 (19)
C28	0.067 (3)	0.057 (2)	0.105 (4)	−0.012 (2)	0.015 (3)	−0.007 (2)
C29	0.076 (3)	0.068 (3)	0.076 (3)	−0.002 (2)	0.009 (2)	0.009 (2)
C30	0.067 (2)	0.072 (3)	0.062 (2)	−0.0066 (19)	−0.0073 (19)	0.010 (2)
C31	0.0435 (16)	0.0431 (17)	0.064 (2)	0.0003 (13)	−0.0067 (15)	−0.0110 (15)
C32	0.0489 (17)	0.0456 (18)	0.064 (2)	0.0034 (14)	−0.0162 (15)	−0.0137 (15)
C33	0.0482 (17)	0.053 (2)	0.054 (2)	−0.0059 (14)	−0.0079 (14)	−0.0057 (15)
C34	0.064 (2)	0.051 (2)	0.070 (2)	−0.0070 (17)	−0.0073 (19)	−0.0102 (18)
C35	0.129 (4)	0.124 (5)	0.070 (3)	−0.039 (3)	−0.012 (3)	−0.051 (3)
C36	0.157 (6)	0.130 (6)	0.129 (5)	0.008 (5)	−0.065 (5)	−0.053 (5)
C37	0.171 (7)	0.100 (5)	0.143 (7)	0.008 (5)	−0.040 (6)	−0.056 (5)
C38	0.133 (5)	0.060 (3)	0.146 (6)	0.016 (3)	−0.023 (5)	−0.025 (4)
C39	0.097 (3)	0.072 (3)	0.079 (3)	0.005 (2)	−0.023 (3)	−0.009 (2)

### Geometric parameters (Å, °)

**Table:**

Pb1—O1	2.957 (8)	C8—H8	0.9300
Pb1—O1'	2.866 (9)	C9—C10	1.369 (6)
Pb1—O2	2.631 (3)	C9—H9	0.9300
Pb1—O3	2.575 (3)	C10—H10	0.9300
Pb1—O4	2.570 (3)	C11—C12	1.441 (5)
Pb1—N1	2.796 (3)	C13—C14	1.511 (6)
Pb1—N2	2.656 (3)	C14—C15	1.368 (7)
Pb1—N3	2.768 (3)	C14—C19	1.390 (7)
Pb1—N4	2.906 (3)	C15—C16	1.334 (10)
Pb1—O7W	2.965 (7)	C15—H15	0.9300
N1—C1	1.324 (5)	C16—C17	1.361 (14)
N1—C12	1.351 (5)	C16—H16	0.9300
N2—C10	1.332 (5)	C17—C18	1.346 (14)
N2—C11	1.357 (4)	C17—H17	0.9300
N3—C21	1.346 (5)	C18—C19	1.383 (7)
N3—C32	1.349 (4)	C18—H18	0.9300
N4—C30	1.319 (5)	C19—H19	0.9300
N4—C31	1.352 (5)	C21—C22	1.386 (6)
O1—C13	1.294 (9)	C21—H21	0.9300
O1'—C13	1.285 (9)	C22—C23	1.348 (7)
O2—C13	1.234 (5)	C22—H22	0.9300
O3—C33	1.247 (4)	C23—C24	1.383 (6)
O4—C33	1.249 (4)	C23—H23	0.9300
O5W—H5WA	0.85	C24—C32	1.419 (5)
O5W—H5WB	0.85	C24—C25	1.438 (6)
O6W—H6WA	0.85	C25—C26	1.336 (7)
O6W—H6WB	0.85	C25—H25	0.9300
O7W—H7WA	0.85	C26—C27	1.417 (7)
O7W—H7WB	0.85	C26—H26	0.9300
F1—C19	1.258 (7)	C27—C31	1.406 (5)
F1'—C15	1.232 (11)	C27—C28	1.410 (7)
F2—F2'	0.578 (12)	C28—C29	1.335 (7)
F2—C39	1.313 (10)	C28—H28	0.9300
F2'—C39	1.344 (8)	C29—C30	1.393 (6)
C1—C2	1.373 (6)	C29—H29	0.9300
C1—H1	0.9300	C30—H30	0.9300
C2—C3	1.334 (7)	C31—C32	1.445 (5)
C2—H2	0.9300	C33—C34	1.508 (5)
C3—C4	1.411 (7)	C34—C39	1.350 (6)
C3—H3	0.9300	C34—C35	1.450 (7)
C4—C12	1.416 (5)	C35—C36	1.387 (8)
C4—C5	1.419 (7)	C35—H35	0.9300
C5—C6	1.331 (7)	C36—C37	1.354 (10)
C5—H5	0.9300	C36—H36	0.9300
C6—C7	1.422 (6)	C37—C38	1.335 (10)
C6—H6	0.9300	C37—H37	0.9300
C7—C8	1.385 (6)	C38—C39	1.388 (8)
C7—C11	1.410 (5)	C38—H38	0.9300
C8—C9	1.364 (7)		
			
O4—Pb1—O3	50.53 (9)	N2—C10—C9	124.4 (4)
O4—Pb1—O2	82.50 (9)	N2—C10—H10	117.8
O3—Pb1—O2	132.87 (9)	C9—C10—H10	117.8
O4—Pb1—N2	77.43 (9)	N2—C11—C7	122.0 (3)
O3—Pb1—N2	93.60 (10)	N2—C11—C12	118.7 (3)
O2—Pb1—N2	77.32 (10)	C7—C11—C12	119.3 (3)
O4—Pb1—N3	70.63 (9)	N1—C12—C4	122.4 (4)
O3—Pb1—N3	84.91 (10)	N1—C12—C11	118.8 (3)
O2—Pb1—N3	74.60 (9)	C4—C12—C11	118.8 (4)
N2—Pb1—N3	139.58 (9)	O2—C13—O1'	123.1 (5)
O4—Pb1—N1	115.89 (10)	O2—C13—O1	120.5 (5)
O3—Pb1—N1	84.61 (10)	O2—C13—C14	119.6 (3)
O2—Pb1—N1	125.93 (10)	O1'—C13—C14	115.0 (5)
N2—Pb1—N1	60.37 (9)	O1—C13—C14	117.9 (5)
N3—Pb1—N1	158.10 (10)	C15—C14—C19	114.9 (5)
O4—Pb1—O1'	129.75 (19)	C15—C14—C13	124.3 (5)
O3—Pb1—O1'	175.83 (19)	C19—C14—C13	120.8 (4)
O2—Pb1—O1'	47.27 (19)	F1'—C15—C16	116.7 (9)
N2—Pb1—O1'	90.5 (2)	F1'—C15—C14	116.0 (8)
N3—Pb1—O1'	91.4 (2)	C16—C15—C14	127.2 (8)
N1—Pb1—O1'	98.3 (2)	C16—C15—H15	116.4
O4—Pb1—N4	107.21 (9)	C14—C15—H15	116.4
O3—Pb1—N4	76.18 (10)	C15—C16—C17	115.7 (9)
O2—Pb1—N4	122.03 (9)	C15—C16—H16	122.2
N2—Pb1—N4	160.24 (9)	C17—C16—H16	122.2
N3—Pb1—N4	57.40 (9)	C18—C17—C16	122.0 (9)
N1—Pb1—N4	101.34 (10)	C18—C17—H17	119.0
O1'—Pb1—N4	100.2 (2)	C16—C17—H17	119.0
O4—Pb1—O1	126.25 (16)	C17—C18—C19	120.5 (8)
O3—Pb1—O1	171.29 (17)	C17—C18—H18	119.8
O2—Pb1—O1	45.79 (16)	C19—C18—H18	119.8
N2—Pb1—O1	77.69 (18)	F1—C19—C18	119.0 (6)
N3—Pb1—O1	101.79 (18)	F1—C19—C14	121.0 (4)
N1—Pb1—O1	90.83 (17)	C18—C19—C14	119.7 (6)
O1'—Pb1—O1	12.8 (2)	C18—C19—H19	120.1
N4—Pb1—O1	112.07 (18)	C14—C19—H19	120.1
O4—Pb1—O7W	176.63 (15)	N3—C21—C22	122.6 (4)
O3—Pb1—O7W	128.7 (2)	N3—C21—H21	118.7
O2—Pb1—O7W	98.0 (2)	C22—C21—H21	118.7
N2—Pb1—O7W	105.94 (17)	C23—C22—C21	119.8 (4)
N3—Pb1—O7W	106.24 (17)	C23—C22—H22	120.1
N1—Pb1—O7W	66.54 (18)	C21—C22—H22	120.1
O1'—Pb1—O7W	50.7 (3)	C22—C23—C24	119.9 (4)
N4—Pb1—O7W	69.71 (18)	C22—C23—H23	120.0
O1—Pb1—O7W	55.1 (2)	C24—C23—H23	120.0
C1—N1—C12	117.0 (4)	C23—C24—C32	118.0 (4)
C1—N1—Pb1	124.3 (3)	C23—C24—C25	123.2 (4)
C12—N1—Pb1	118.5 (2)	C32—C24—C25	118.8 (4)
C10—N2—C11	117.4 (3)	C26—C25—C24	121.2 (4)
C10—N2—Pb1	119.1 (2)	C26—C25—H25	119.4
C11—N2—Pb1	123.4 (2)	C24—C25—H25	119.4
C21—N3—C32	117.8 (3)	C25—C26—C27	121.5 (4)
C21—N3—Pb1	116.7 (3)	C25—C26—H26	119.3
C32—N3—Pb1	125.4 (2)	C27—C26—H26	119.3
C30—N4—C31	116.7 (3)	C31—C27—C28	116.2 (4)
C30—N4—Pb1	122.8 (3)	C31—C27—C26	120.2 (4)
C31—N4—Pb1	120.5 (2)	C28—C27—C26	123.6 (4)
C13—O1—Pb1	84.5 (4)	C29—C28—C27	121.2 (4)
C13—O1'—Pb1	88.6 (5)	C29—C28—H28	119.4
C13—O2—Pb1	101.0 (2)	C27—C28—H28	119.4
C33—O3—Pb1	92.0 (2)	C28—C29—C30	117.8 (4)
C33—O4—Pb1	92.2 (2)	C28—C29—H29	121.1
H5WA—O5W—H5WB	103.4	C30—C29—H29	121.1
H6WA—O6W—H6WB	114.0	N4—C30—C29	125.0 (4)
Pb1—O7W—H7WA	79.7	N4—C30—H30	117.5
Pb1—O7W—H7WB	125.1	C29—C30—H30	117.5
H7WA—O7W—H7WB	117.9	N4—C31—C27	123.2 (4)
F2'—F2—C39	80.5 (15)	N4—C31—C32	118.0 (3)
F2—F2'—C39	74.4 (14)	C27—C31—C32	118.8 (3)
N1—C1—C2	124.8 (5)	N3—C32—C24	121.8 (4)
N1—C1—H1	117.6	N3—C32—C31	118.6 (3)
C2—C1—H1	117.6	C24—C32—C31	119.5 (3)
C3—C2—C1	118.9 (5)	O3—C33—O4	123.2 (3)
C3—C2—H2	120.6	O3—C33—C34	119.0 (3)
C1—C2—H2	120.6	O4—C33—C34	117.8 (3)
C2—C3—C4	120.4 (4)	C39—C34—C35	118.9 (4)
C2—C3—H3	119.8	C39—C34—C33	124.4 (4)
C4—C3—H3	119.8	C35—C34—C33	116.6 (4)
C3—C4—C12	116.5 (4)	C36—C35—C34	113.7 (6)
C3—C4—C5	123.4 (4)	C36—C35—H35	123.2
C12—C4—C5	120.1 (4)	C34—C35—H35	123.2
C6—C5—C4	120.4 (4)	C37—C36—C35	125.0 (7)
C6—C5—H5	119.8	C37—C36—H36	117.5
C4—C5—H5	119.8	C35—C36—H36	117.5
C5—C6—C7	122.5 (4)	C38—C37—C36	121.1 (6)
C5—C6—H6	118.8	C38—C37—H37	119.4
C7—C6—H6	118.8	C36—C37—H37	119.4
C8—C7—C11	117.3 (4)	C37—C38—C39	116.6 (6)
C8—C7—C6	123.8 (4)	C37—C38—H38	121.7
C11—C7—C6	119.0 (4)	C39—C38—H38	121.7
C9—C8—C7	120.8 (4)	F2—C39—C34	119.5 (6)
C9—C8—H8	119.6	F2'—C39—C34	116.9 (5)
C7—C8—H8	119.6	F2—C39—C38	113.7 (6)
C8—C9—C10	118.0 (4)	F2'—C39—C38	117.8 (6)
C8—C9—H9	121.0	C34—C39—C38	124.6 (5)
C10—C9—H9	121.0		

### Hydrogen-bond geometry (Å, °)

**Table:**

*D*—H···*A*	*D*—H	H···*A*	*D*···*A*	*D*—H···*A*
O5W—H5WA···O2	0.85	1.98	2.796 (4)	162
O5W—H5WB···O6W	0.85	1.97	2.757 (5)	153
O6W—H6WA···O5W^i^	0.85	1.98	2.809 (6)	163
O6W—H6WB···O4	0.85	1.97	2.818 (3)	175
O6W—H6WA···O5W^i^	0.85	1.98	2.809 (6)	163
O7W—H7WA···O1'	0.85	1.98	2.496 (5)	118
O7W—H7WB···O1'^ii^	0.85	1.99	2.565 (2)	124
C8—H8···F1^iii^	0.93	2.54	3.310 (7)	141
C30—H30···F1'^ii^	0.93	2.50	3.032 (12)	115
C29—H29···O3^iv^	0.93	2.46	3.311 (5)	153

Symmetry codes:  (i) −*x*+2, −*y*+2, −*z*; (ii) −*x*+1, −*y*+2, −*z*+1; (iii) *x*+1, *y*, *z*; (iv) −*x*+1, −*y*+1, −*z*+1.

## References

[bb1] Bruker (2007). *SMART* and *SAINT* Bruker AXS Inc., Madison, Wisconsin, USA.

[bb2] Sheldrick, G. M. (1996). *SADABS* University of Göttingen, Germany.

[bb3] Sheldrick, G. M. (2008). *Acta Cryst.* A**64**, 112–122. 10.1107/S0108767307043930 18156677

[bb4] Zhang, B.-S. (2004). *Z. Kristallogr. New Cryst. Struct.* **219**, 483–484.

[bb5] Zhang, B.-S. (2005). *Z. Kristallogr. New Cryst. Struct.* **220**, 73–74.

[bb6] Zhang, B.-S. (2006*a*). *Acta Cryst.* E**62**, m2645–m2647.

[bb7] Zhang, B.-S. (2006*b*). *Z. Kristallogr. New Cryst. Struct.* **221**, 191–194.

[bb8] Zhang, B. S. (2006*c*). *Z. Kristallogr. New Cryst. Struct.* **221**, 355–356.

[bb9] Zhang, B.-S., Zeng, X.-R., Yu, Y.-Y., Fang, X.-N. & Huang, C.-F. (2005). *Z. Kristallogr. New Cryst. Struct.* **220**, 75–76.

